# Molecular identification of *Mazama* species (Cervidae: Artiodactyla) from natural history collections

**DOI:** 10.1590/1678-4685-GMB-2019-0008

**Published:** 2020-03-23

**Authors:** Aline Meira Bonfim Mantellatto, Susana González, José Maurício Barbanti Duarte

**Affiliations:** 1 Universidade Estadual Paulista “Júlio de Mesquota Filho” (UNESP), Faculdade de Ciências Agrárias e Veterinárias, Núcleo de Pesquisa e Conservação de Cervídeos, Jaboticabal, SP, Brazil.; 2 Universidade Federal do Sul da Bahia, Centro de Formação em Ciências Ambientais, Laboratório de Ecologia e Conservação Marinha, Campus Sosígenes Costa, Porto Seguro, BA, Brazil.; 3 Instituto de Investigaciones Biológicas Clemente Estable, Departamento de Biodiversidad y Genética, Montevideo, Uruguay.

**Keywords:** Cytochrome b, deer, DNA, mammal, morphological taxonomy

## Abstract

Natural history museum collections constitute an invaluable patrimony of biological diversity for analysing the taxa distribution and evolution. However, it is very common to discover taxonomic misidentification in museum collections based on incorrect data. The aim of this research was to identify brocket deer species (*Mazama* genus) using molecular markers. We collected 199 samples, performed DNA extraction and species identification using a specific mitochondrial marker based on a fragment of cytochrome b (*Cytb*) for Neotropical deer. We achieved the amplification and sequencing of 77 specimens and verified that 26% of the skulls were wrongly identified. Moreover, in the museum collections 57% of the specimens were only identified as *Mazama* sp, and we were able to identify them by molecular methods to the species level. Our findings clearly demonstrate the importance of integrating molecular analyses to identify *Mazama* species, since using only external morphology can result in a high probability of errors. We recommend the selection of non-convergent morphological characters, which together with the use of DNA collected from museum specimens should contribute to more accurate taxonomic identifications.

## Introduction

Biological museum collections house millions of specimens worldwide (Wandeler *et al.*, 2007), representing the diversity of plants and animals that exist in the world, and are the main source for consultation and the development of studies involving geographical and temporal distribution of living beings (Wandeler *et al.*, 2007; Goodwin *et al.*, 2015). The information contained on the labels of each specimen is always assumed to be correct, even though errors in taxonomic identification of specimens are commonly known to occur (Goodwin *et al.*, 2015). The correct delimitation of boundaries between species is crucial to our knowledge of the diversity of life, as they determine whether or not the organisms in question are members of the same entity (Dayrat, 2005). In addition, the misidentification might detrimentally impact species conservation (George and Mayden, 2005; Gutiérrez and Helgen, 2013; Gippoliti *et al.*, 2017).

Few studies have been designed to verify the extent of specimen misidentifications in museum collections. For example, Goodwin *et al.* (2015), evaluated the accuracy of names associated with plant species from 40 herbariums in 21 countries, and their results showed that at least 58% of the specimens were misidentified. One of the possible reasons of high identification errors is the decrease in the formation of new taxonomists, while the number of specimens in collections increases over time (Goodwin *et al.*, 2015). Indeed, the profusion of identification errors associated with the names present in museum classifications give rise to taxonomic problems that affect hypotheses and ideas, and represents a deep practical problem that affects our knowledge about nature (Bortolus, 2008).

The genus *Mazama* Rafinesque 1817 (brocket deer) is a taxon that encompasses several species with convergent morphology that are very difficult to distinguish (Allen, 1915). The genus is considered one of the most remarkable and surprising cases of morphological convergence among mammals (Gilbert *et al.*, 2006; Duarte *et al.*, 2008, González *et al.*, 2010), with doubts remaining regarding the evolutionary relationships between the species described up to now. Previous morphological analyses performed by Merino *et al.* (2005) considered that *Mazama* is monophyletic. However several studies using several molecular analyses, such as isoenzymes (Smith *et al.*, 1986), mitochondrial and nuclear markers (Gilbert *et al.*, 2006, Duarte *et al.*, 2008; Hassanin *et al.*, 2012; Escobedo-Morales *et al.*, 2016; Heckeberg *et al.*, 2016; Gutiérrez *et al.*, 2017) revealed the genus is polyphyletic.

Recent studies on species of Neotropical deer have further explored a combination of data on skull morphometrics and sequences of the cytochrome b (*Cytb*) gene (Gutiérrez *et al.*, 2015, 2017). However, taxonomic misidentification of species of the genus *Mazama* based on incorrect data might still be present in natural history collections, propagating misidentifications of species names.

Duarte *et al.* (2008) described one of the most amazing cases of morphological convergent evolution and cryptic species system in mammals, where brocket deer with very similar external morphologies showed high levels of molecular and cytogenetic differentiation. At least eight ancestral forms of deer invaded South America since the late Pliocene (2.5–3 MYA), and members of the red brockets had an independent early explosive diversification soon after their ancestor arrived there, giving rise to a number of morphologically cryptic species (Duarte *et al.*, 2008). Taxonomic revision of this group based on cytogenetic data have proven to be more useful in comparison with morphological approaches in recognizing new cryptic species of red brockets from Mexico *(Mazama temama*; Groves and Grubb, 1987), and from Brazil (*Mazama bororo*; Duarte and Jorge, 2003).

Morphological and morphometric analyses have been performed on museum collections in Brazil, indicating high intraspecific polymorphism, hindering the process of discrimination between brocket deer species (Rossi RV, personal communication). This happens due to high levels of homoplasy in the morphological characters of *Mazama* (Duarte *et al.*, 2008; González *et al.*, 2010). Thus, given the enormous potential of generating large amounts of DNA sequence data from museum specimens, sequencing tech-nologies offer one of the most promising approaches to resolve discrepancies in taxonomy (Gutiérrez *et al.*, 2017).

Here, we used molecular markers to elucidate species-level brocket deer identifications in specimens from Brazilian natural history collections, as the genus *Mazama* contains several cryptic species and many doubts remain concerning the evolutionary relationships among the currently recognized species.

## Material and Methods

### Mazama samples

We collected samples from 199 *Mazama* specimens between September and December 2013, which were deposited in 10 Brazilian natural history collections over the past 100 years (Table 1).

**Table 1:** The natural history collections visited, their respective acronyms, locations and number of turbinate bones collected.

Museum	City/State of Museum Location	No. of specimens obtained
Museu de Zoologia da Universidade de São Paulo (MZUSP)	São Paulo, São Paulo	49
Museu Nacional do Rio de Janeiro (MNRJ)	Rio de Janeiro, Rio de Janeiro	23
Museu de História Natural Capão da Imbuia (MHNCI)	Curitiba, Paraná	75
Museu de História Natural Professor Adão José Cardoso (ZUEC)	Campinas, São Paulo	6
Museu de Biologia Professor Mello Leitão (MBML)	Santa Teresa, Espírito Santo	12
Fundação Zoobotânica do Rio Grande do Sul (FZB)	Porto Alegre, Rio Grande do Sul	14
Museu Anchieta (MAMM)	Porto Alegre, Rio Grande do Sul	3
Museu de Zoologia da Pontifícia Universidade Católica do Rio Grande do Sul (PUC-RS)	Porto Alegre, Rio Grande do Sul	4
Museu de Zoologia da Universidade Federal da Paraíba (UFPB)	João Pessoa, Paraíba	7
Museu de Zoologia da Universidade Federal de Pernambuco (UFPE)	Recife, Pernambuco	6

We collected approximately 180 mg of turbinate bone fragments of skulls from specimens labeled as genus *Mazama* (Wiseley *et al.*, 2004). We used long-handled tweezers to remove these pieces of bone, and cleaned these instruments with bleach every time before handling the next specimen. The bone fragments were stored in sterilized 50 mL plastic tubes and identified with the museum’s acronym and the collection number of the respective skull.

### DNA extraction, amplification and sequencing

DNA extraction was performed using the protocol proposed by González *et al.* (2015). In order to minimize the risks of contamination and to ensure the reliability of the results, negative controls were used in all DNA extractions and were quantified in a NanoDrop 2000 spectrophotometer (Thermo Fisher Scientific, Delaware, USA). In addition, at all stages, we used filter tips and disposable gloves.

Polymerase chain reactions (PCR) were performed in a laminar flow cabinet, accompanied by negative controls that were also submitted to the amplification step to confirm the absence of contaminating DNA in the reactions. We used specific primers (González *et al.*, 2009) to amplify a 224 bp region of the mitochondrial cytochrome b gene (IDMAZ224L and IDMAZ H): forward sequence, 5’ CATCCGACACAATAACAGCA 3’; reverse sequence, 5’ TCCTACGAATGCTGTGGCTA 3’. Amplifications were performed in a real-time thermal cycler (Rotor Gene - Qiagen Inc, Texas, USA), testing aliquots of extracted DNA of 10 ng/μL, 30 ng/μL, 50 ng/μL and 100 ng/μL (Lozier and Cameron, 2009; Tex *et al.*, 2010; Woide *et al.*, 2010; Allentoft *et al.*, 2011; Andersen *et al.*, 2011; Paplinska *et al.*, 2011). The best result was achieved using aliquots at 10 ng/μL. We then used these aliquots to amplify the 199 samples. The final volume of the amplification reactions was 20 μL, consisting of: 1 μL of the SensiFast^TM^ HRM kit, 0.8 μM of each primer, 0.3 μL of BSA, 10 ng/μL of DNA and 6.9 μL of water. After initial denaturation at 95 °C for 2 min, a protocol with three different profiles was run: 95 °C for 5 s, 55 °C for 10 s (10 cycles), 54 °C for 10 s (15 cycles), 53 °C for 10 s (15 cycles) and 72 °C for 20 s, followed by a final holding step for 5 min, before a melting curve analysis was run.

After purifying the samples according to the protocol described by Dorado-Pérez (2008), both strands (forward and reverse) were sequenced using an automated sequencer (3730XL DNA Analyzer, Applied Biosystems, California, USA). In order to confirm the results obtained, the amplifications and DNA sequencing reactions for each sample were performed twice in different laboratories.

### Molecular data analysis

We visually analysed the quality of the sequences using PHRED software, included in the package CODON CODE ALIGNER version 6.0.2. Sequences with less than 50 bases with PHRED 20 were excluded. Sequence alignment was performed using CLUSTALW (Thompson *et al.*, 1994), included in BIOEDIT version 7.2.5 (Hall, 1999). To identify the species of each specimen, the sequences obtained were compared with the reference sequences of *Cytb* of 985 bp downloaded from GenBank, using the BLAST tool (blast.ncbi.nlm.nih.gov). The sequences were analysed in CIPRES SCIENCE GATEWAY (Miller *et al.*, 2010) using the JMODELTEST version 2 (Darriba *et al.*, 2012) to determine which evolutionary model best fit the gene under analysis. The criterion used to select the best model was the Bayes Information Criteria (BIC), with the Hasegawa, Kishino and Yano (HKY) model + G selected as the best fit.

BEAST software version 1.8.1 (Drummond *et al.*, 2012) was used for phylogenetic tree inference based on Bayesian analysis, using 35,000,000 generations. This procedure grouped sample sequences from museum specimens with sequences of *Mazama* species in such a way that identification of the species of each specimen was the most robust. We rooted the tree using a sequence of *Rangifer tarandus*. A 25% burn-in was applied. The convergence among runs was verified using TRACER version 1.6, and only effective sample size (ESS) results higher than 200 were accepted. The resulting trees were condensed in the TREEANNOTATOR software, and visualization of the trees was achieved using FIGTREE, version 1.3.1 (Rambaut, 2010).

Sequences of specimens of *Mazama americana* (Erxleben, 1777), *Mazama nana* (Hensel, 1872), *Mazama bororo* (Duarte, 1996), *Mazama gouazoubira* (Fischer, 1814) and *Rangifer tarandus* (Linnaeus, 1758) used for the phylogenetic tree inference are available in GenBank or the NUPECCE sequence database (Table 2).

**Table 2:** Sequences used for phylogenetic tree inference, detailing the sample origin as recorded from museum label collections with sequences from known identification and GenBank access number.

Species	Specimen identification	GenBank Access	Collection location
*Mazama americana*	T40	DQ789224.2	Pará
	T43	MG786262	Pará
	T110	DQ789201.2	Paraná
	T205	DQ789215.2	Paraná
*Mazama bororo*	T71	DQ789231.2	São Paulo
	T72	MG786263	São Paulo
	T338	MG786261	Paraná (in captivity)
	Msg54	DQ789187.2	———
*Mazama nana*	T2	DQ789214.2	Paraguay
	T53	DQ789227.2	Paraná (in captivity)
	T185	DQ789210.2	Paraná
*Mazama gouazoubira*	T112	DQ789202.2	São Paulo
*Rangifer tarandus*		KX067075.1	———

——- indicates an unknown geographic location

## Results

**Figure 1:**
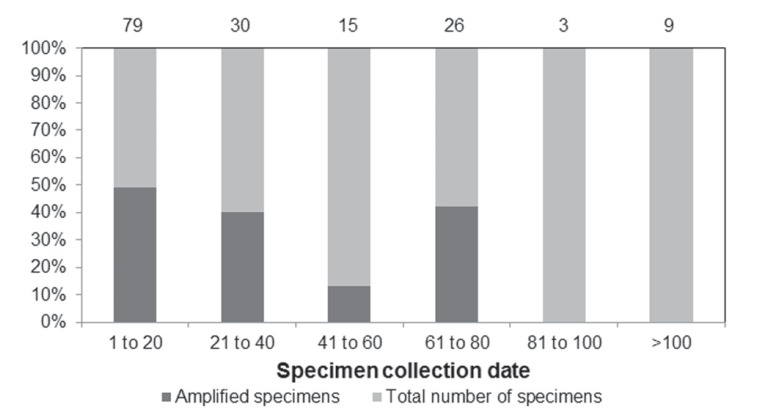
DNA amplification success (dark grey) according to the collection date of 162 specimens from natural history collections. The numbers above the bars indicate the total number of specimens present in each time period and the numbers below the bars indicate the age of the samples.

Following the identification on the label of each skull selected, we collected samples from 39 specimens of *M. americana*, one of *Mazama rufa* (Illiger, 1815), synonymous with *M. americana*, 58 of *M. gouazoubira*, 11 of *Mazama simplicicornis* (Illiger, 1815), synonymous with *M. gouazoubira*, two of *M. nana*, six of *Mazama rufina* (Bourcier and Pucheran 1852), synonymous with *M. nana*, and 82 identified as *Mazama* sp, resulting in 199 specimens total. Our samples included both recent specimens (1-20 years old) and very old ones (> 81 years old). However, information on specimen age on the respective label was only referenced in 162 specimens (Figure 1). The amplification success rate was 49.3% (Figure 1). The mean DNA quantified in all samples was 443 ng/μL. Amplification success and specimen identification was 39%.

**Figure 2:**
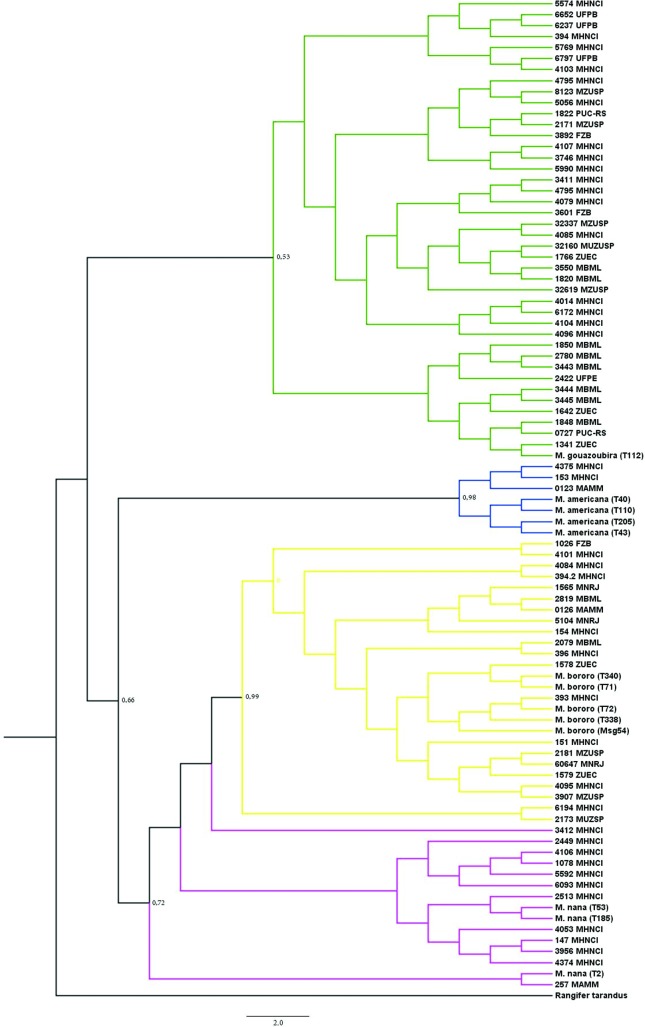
Phylogenetic tree inferred from Bayesian analysis, demonstrating the relationships between recent DNA samples and museum collections within the genus *Mazama*, identified by a fragment of the mitochondrial *Cytb*. Reference samples are represented by the species name and identification code. Museum specimens are represented by their registration number and museum acronym. The colours indicate the four species identified in the museum specimens (*M. americana*, blue; *M. nana*, pink; *M. bororo*, yellow; *M. gouazoubira*, green).

Based on specimen grouping in the phylogenetic analysis, we identified three skulls of *M. americana*, 41 of *M. gouazoubira*, 12 of *M. nana* and 21 of *M. bororo* with high statistical support (Figure 2, Table S1). Compared with the information indicated on labels, our molecular identification highlighted differences in identification (Table 3). We determined that skulls from *M. gouazoubira* and *M. nana* had the lowest error rate. In contrast, skulls of *M. americana* had the highest error rate (Table 3).

**Table 3:** Comparison among the morphological information of 77 specimens identified with *Cytb* from Brazilian museum collections.

Museum identification		Molecular identification			
		*M. gouazoubira*	*M. americana*	*M. nana*	*M. bororo*
*M. gouazoubira*	21	11	1	2	7
*M. americana*	7	3	0	1	3
*M. nana*	6	0	0	4	2
*Mazama* sp.	43	27	2	5	9
Total	77	41	3	12	21

## Discussion

Our results show that molecular techniques are useful tools for increasing the reliability of *Mazama* identification in natural history collections. Further, we highlight that identification errors could propagate unrealistic names of the species, probably because of poorly made taxonomic revisions, based on a small sample and ignoring geographic variation, obscuring the diversity in *Mazama*.

Given that we worked with fragments of turbinate bones, which naturally have low DNA quality compared with fresh samples (e.g. blood), we expected to find low rates of success in DNA extraction, amplification and sequencing. DNA extracted from historical material is expected to be highly degraded and highly diluted, similar to DNA from non-invasive sampling (Taberlet *et al.*, 1999). Variation exists in the preservation of DNA in historical specimens due to specimen age and type of museum preparation (McDonough *et al.*, 2018), and also due to degradation by microorganisms, as well as oxidative and hydrolytic lesions that can further negatively affect DNA quality (Hall *et al.*, 1997; Pääbo *et al.*, 2004; Rohland *et al.*, 2004; Tang 2006). In addition, several *post-mortem* processes cause DNA damage, and these processes are more significant for ancient DNA (Rambaut *et al.*, 2009). Nonetheless, we were able to use molecular techniques to improve *Mazama* species identification and highlight the importance of this tool for correct identification in natural history collections.

### Molecular analysis

We obtained a very surprising result, showing that none of the skulls identified as *M. americana* were in fact *M. americana*. Indeed, *M. americana* is actually composed of several species, i.e. it is a complex of species (Duarte *et al.*, 2008; Abril *et al.*, 2010), which further complicates its morphologically identification. Another curious result was that 21 samples molecularly identified as *M. bororo* were not labelled as such in any of the museums. This can be explained by the recent description of this species (Duarte and Giannoni, 1996) and the fact that *Mazama* species are morphologically quite similar, further complicating taxonomic identification (Duarte, 1996). The absence of statistically significant morphometric differences between the skulls of *M. bororo* and *M. americana* was observed by Rossi RV (personal communication). Our molecular analysis indicated that the rate of misidentification errors for every taxon based on morphological characters was 26%. Very similar results were also reported by Moraes-Barros *et al.* (2011), who reviewed morphological and molecular data from museum collections of two species of sloths and observed identification errors attributed to the similarity in coat colouration.

Our results show the difficulty of correctly identifying brocket deer species based solely on cranial morphological characters. However, the taxonomy of Neotropical deer has been established almost entirely on the basis of descriptive morphological data, without the use of explicit phylogenetic methods (Gutiérrez *et al.*, 2017). Similar to what we found, Duarte *et al.* (2008) demonstrated high levels of phylogenetic distinction between various forms of morphologically similar brocket deer, highlighting that phylogenetic relationships based on external morphological characters, such as pelage colouration and body size and shape, are problematic because of extensive homoplasy. In this case, the *Cytb* PCR protocol was extremely useful, since we were also able to identify 57% of our samples labelled as *Mazama* sp. (Table S1).

Our findings clearly demonstrate the importance of integrating molecular data to identify *Mazama* species, as using external morphology alone can result in a high rate of errors, given the several cryptic species in the genus *Mazama* (Duarte *et al.*, 2008). We also demonstrated the advantages of performing museum identifications using molecular markers, like *Cytb*. This method is economically advantageous, and only a very small amount of bone fragments is needed to obtain DNA from each specimen, minimally impacting the conservation of these collections. Further, it allows to update taxonomic identifications and better understand the evolutionary relationships of the taxa. We also recommend the selection of non-convergent morphological characters, which together with the use of museum DNA should contribute to more accurate taxonomic identifications.

## Supplementary Material

The following online material is available for this article:

Table S1 Molecular identification of DNA samples collections in museums based on 224 bp fragment of the mitochondrial *Cytb*.

*Associate Editor: Fabrício Rodrigues dos Santos*
