# Supplementary material for: Molecular identification of Mazama species (Cervidae: Artiodactyla) from natural history collections
Source: Genet Mol Biol. 2020 Mar 23;43(2):e20190008. doi: 10.1590/1678-4685-GMB-2019-0008 (PMC7197991; doi:10.1590/1678-4685-GMB-2019-0008)
Supplement: Supplementary file 1 [file 1415-4757-GMB-43-2-e20190008-suppl1.pdf]

Supplementary Material "Molecular identification of *Mazama* species (Cervidae: Artiodactyla) from natural history collections"

Table S1 - Molecular identification of DNA samples collections in museums based on 224 bp fragment. of the mitochondrial *Cytb*.

Museum ID	Species: museum ID	Species: molecular ID	GenBank Access	Geographic location	Year collected
3907 MZUSP	*M. americana*	*M. bororo*	MG786264	SP	1934
32160 MZUSP	*M. gouazoubira*	*M.gouazoubira*	MG786298	SP	-------------
2181 MZUSP	*M. gouazoubira*	*M. bororo*	MG786277	SP	-------------
8123 MZUSP	*M. gouazoubira*	*M. gouazoubira*	MG786299	MA	1955
2171 MZUSP	*M. americana*	*M. gouazoubira*	MG786300	SP	2006
2173 MZUSP	*M. gouazoubira*	*M. bororo*	MG786340	SP	-------------
32337 MZUSP	*M. gouazoubira*	*M. gouazoubira*	MG786301	SP	-------------
32619 MZUSP	*M. gouazoubira*	*M. gouazoubira*	MG786302	SP	-------------
6237 UFPB	*Mazama* sp.	*M. gouazoubira*	MG786303	PB	2011
6652 UFPB	*M. gouazoubira*	*M. gouazoubira*	MG786331	PE	2012
6797 UFPB	*Mazama* sp.	*M. gouazoubira*	MG786304	PB	2012
2422 UFPE	*M. gouazoubira*	*M.gouazoubira*	MG786305	MA	2000
0123 MAMM	*Mazama sp.*	*M.americana*	MG786295	RS	-------------
0126 MAMM	*Mazama sp.*	*M. bororo*	MG786265	RS	-------------
257 MAMM	*M. americana*	*M.nana*	MG786283	RS	-------------
1341 ZUEC	*Mazama sp.*	*M.gouazoubira*	MG786306	SP	-------------
1578 ZUEC	*Mazama sp.*	*M.bororo*	MG786278	SP	1979
1579 ZUEC	*Mazama sp.*	*M.bororo*	MG786279	SP	1979
1642 ZUEC	*Mazama sp.*	*M.gouazoubira*	MG786307	SP	1993
1766 ZUEC	*Mazama sp.*	*M.gouazoubira*	MG786308	SP	1994
1820 MBML	*M. americana*	*M.gouazoubira*	MG786309	ES	1993
1848 MBML	*M. gouazoubira*	*M.gouazoubira*	MG786332	ES	1996
1850 MBML	*M. americana*	*M.gouazoubira*	MG786333	ES	1996
2079 MBML	*M. americana*	*M.bororo*	MG786266	ES	1994
2780 MBML	*Mazama sp.*	*M.gouazoubira*	MG786310	ES	2005
2819 MBML	*M. americana*	*M. bororo*	MG786282	ES	2008
3443 MBML	*Mazama sp.*	*M.gouazoubira*	MG786311	ES	-------------
3444 MBML	*Mazama sp.*	*M.gouazoubira*	MG786312	ES	2010
3445 MBML	*Mazama sp.*	*M.gouazoubira*	MG786313	ES	2010
3500 MBML	*Mazama sp.*	*M.gouazoubira*	MG786334	ES	-------------
0727 PUC-RS	*Mazama sp.*	*M.gouazoubira*	MG786314	RS	-------------
1822 PUC-RS	*M. gouazoubira*	*M.gouazoubira*	MG786315	RS	2012
1565 MNRJ	*Mazama sp.*	*M. bororo*	MG786267	MG	1934
5104 MNRJ	*M. gouazoubira*	*M.bororo*	MG786268	GO	1936
60647 MNRJ	*M. gouazoubira*	*M.bororo*	MG786269	BA	1956
3892 FZB	*M. gouazoubira*	*M.gouazoubira*	MG786316	RS	1992
1026 FZB	*M. gouazoubira*	*M.bororo*	MG786270	RS	1983

Museum ID	Species: museum ID	Species: molecular ID	GenBank Access	Geographic location	Year collected
3601 FZB	*Mazama sp.*	*M.gouazoubira*	MG786317	RS	2012
4104 MHNCI	*Mazama sp.*	*M,gouazoubira*	MG786318	PR	1996
4096 MHNCI	*Mazama sp.*	*M.gouazoubira*	MG786319	PR	1999
4103 MHNCI	*Mazama sp.*	*M.gouazoubira*	MG786320	PR	1999
4095 MHNCI	*M. rufina*	*M.bororo*	MG786280	PR	2001
4375 MHNCI	*Mazama sp.*	*M.americana*	MG786294	PR	2004
4107 MHNCI	*Mazama sp.*	*M.gouazoubira*	MG786321	PR	1997
4374 MHNCI	*M. nana*	*M.nana*	MG786284	PR	2004
1078 MHNCI	*M. rufina*	*M.nana*	MG786285	PR	1987
5574 MHNCI	*Mazama sp.*	*M. gouazoubira*	MG786322	PR	2004
4085 MHNCI	*M. gouazoubira*	*M. gouazoubira*	MG786335	PR	1996
4795 MHNCI	*Mazama sp.*	*M.gouazoubira*	MG786323	PR	1996
5580 MHNCI	*Mazama sp.*	*M.gouazoubira*	MG786324	PR	2003
5592 MHNCI	*Mazama sp.*	*M.nana*	MG786290	PR	-------------
4101 MHNCI	*M. gouazoubira*	*M.bororo*	MG786271	PR	2002
5056 MHNCI	*Mazama sp.*	*M.gouazoubira*	MG786325	PR	1996
3746 MHNCI	*Mazama sp.*	*M.gouazoubira*	MG786326	PR	1997
394 MHNCI	*Mazama sp.*	*M.gouazoubira*	MG786327	PR	1949
3412 MHNCI	*Mazama sp.*	*M.nana*	MG786286	PR	1995
151 MHNCI	*M. simplicicornis*	*M. bororo*	MG786272	PR	1945
393 MHNCI	*Mazama sp.*	*M.bororo*	MG786281	PR	1950
396 MHNCI	*Mazama sp.*	*M.bororo*	MG786273	PR	1950
4084 MHNCI	*Mazama sp.*	*M.bororo*	MG786274	PR	1996
5990 MHNCI	*Mazama sp.*	*M.gouazoubira*	MG786330	PR	2007
394.2 MHNCI	*Mazama sp.*	*M. bororo*	MG786275	PR	1949
4106 MHNCI	*Mazama sp.*	*M.nana*	MG786291	PR	1999
147 MHNCI	*M. simplicicornis*	*M.nana*	MG786292	PR	1945
2513 MHNCI	*M. rufina*	*M.nana*	MG786287	PR	1985
5769 MHNCI	*Mazama sp.*	*M.gouazoubira*	MG786337	PR	2005
3411 MHNCI	*Mazama sp.*	*M.gouazoubira*	MG786338	PR	-------------
6172 MHNCI	*Mazama sp.*	*M.gouazoubira*	MG786328	PR	1999
4079 MHNCI	*M. gouazoubira*	*M. gouazoubira*	MG786339	PR	2001
4014 MHNCI	*Mazama sp.*	*M.gouazoubira*	MG786329	PR	1998
3956 MHNCI	*M. gouazoubira*	*M.nana*	MG786293	PR	1999
2449 MHNCI	*Mazama sp.*	*M.nana*	MG786288	PR	1992
153 MHNCI	*M. simplicicornis*	*M.americana*	MG786296	PR	1945
6093 MHNCI	*Mazama sp.*	*M.nana*	MG786297	PR	2008
4053 MHNCI	*Mazama sp.*	*M.nana*	MG786289	PR	2001
154 MHNCI	*M. simplicicornis*	*M.bororo*	MG786276	PR	1944
6194MHNCI	*Mazama sp.*	*M. bororo*	MG786336	PR	2008
